# Supplementary material for: cisDynet: An integrated platform for modeling gene‐regulatory dynamics and networks
Source: Imeta. 2023 Nov 23;2(4):e152. doi: 10.1002/imt2.152 (PMC10989917; doi:10.1002/imt2.152)
Supplement: Supplementary file 1 — Figure S1: Screenshot of the HTML report generated by cisDynet's pre‐processing pipeline. Figure S2: ATAC‐seq classical quality control chart. Figure S3: All public tools used by cisDynet. Figure S4: Schematic diagram of cisDynet constructing a regulatory network. Figure S5: Screenshots from the Genome Browser showing the “problematic” regions identified in Arabidopsis (top) and rice (bottom). Figure S6: Using cisDynet to identify tissue‐specific OCRs. Figure S7: The basic description of the peak‐to‐gene links. [file IMT2-2-e152-s001.docx]

**Supplementary information to:**

Title: **cisDynet: an integrated platform for modeling gene-regulatory dynamics and networks**

Running Title: **cisDynet enables the exploration of the cis-regulatory chromatin dynamics and networks.**

Tao Zhu^#^, Xinkai Zhou^#^, Yuxin You^#^, Lin Wang, Zhaohui He, Dijun Chen^*^

State Key Laboratory of Pharmaceutical Biotechnology, School of Life Sciences, Nanjing University, Nanjing 210023, China;

^#^ These authors contributed equally to this work.

^*^ Correspondence: dijunchen@nju.edu.cn (Dijun Chen)

**Supplementary Figures**

**Figure S1**: Screenshot of the HTML report generated by cisDynet’s pre-processing pipeline.

**Figure S2:** ATAC-seq classical quality control chart.

**Figure S3:** All public tools used by cisDynet.

**Figure S4**: Schematic diagram of cisDynet constructing a regulatory network.

**Figure S5:** Screenshots from the Genome Browser showing the “problematic” regions identified in Arabidopsis (top) and rice (bottom).

**Figure S6:** Using cisDynet to identify tissue-specific OCRs.

**Figure S7:** The basic description of the peak-to-gene links.

**Figure S1** Screenshot of the HTML report generated by cisDynet’s pre-processing pipeline.

**Figure S2** ATAC-seq classical quality control chart. Left: fragment insertion distribution map with patterns of nucleosome-free, mono- and dinucleosome distribution near 100bp, 200bp and 400bp, respectively; right, Tn5 cuts are significantly enriched around the TSSs.

**Figure S3** All public tools used by cisDynet. All the tools can be divided into three main blocks: data pre-processing, data analysis and data visualization.

**Figure S4** Schematic diagram of cisDynet constructing a regulatory network. A transcription factor (TF) is considered to regulate this gene if the footprint of a particular TF is present in the peak associated with this gene.

**Figure S5** Screenshots from the Genome Browser showing the “problematic” regions identified in Arabidopsis (top) and rice (bottom).

**Figure S6** Using cisDynet to identify tissue-specific open chromatin regions (OCRs). (A) PCA plot demonstrating the similarity between different ATAC-seq samples. Data from Diego Calderon *et al.*. (B) Tissue-specific OCRs obtained based on Shannon entropy. (C) The boxplot shows the distribution of specificity measure (SPM) scores in each sample. (D) Heatmap showing OCRs screened by SPM score that are specifically open only in Memory B, Bulk B and Naive B cells. (E) Heatmap showing highly specific OCRs in each sample based on SPM score screening.

**Figure S7** The basic description of the peak-to-gene links. (A) The density plot illustrates the relationship between peak-to-gene distances and correlation coefficients. (B) Distance distribution of all identified reliable peak-to-gene. (C) Distribution of the number of peaks associated with each gene in all identified reliable peak-to-gene. (D) Distribution of the number of genes associated with each peak in all identified reliable peak-to-gene. (E) Of all the reliable peak-to-gene links identified, the dashed line represents the average number of peaks associated with a TF, and the density plot is the average number of peaks associated with a randomly selected (repeated 1000 times) gene. *p*-value was calculated by Z-test. (F) For all peak-gene associations with correlation coefficients greater than 0.6, the dotplot shows the biological pathways enriched for genes with more than 10 peaks.
